# Hippocampal Anatomy Supports the Use of Context in Object Recognition: A Computational Model

**DOI:** 10.1155/2013/294878

**Published:** 2013-05-25

**Authors:** Patrick Greene, Mike Howard, Rajan Bhattacharyya, Jean-Marc Fellous

**Affiliations:** ^1^Graduate Program in Applied Mathematics, University of Arizona, Tucson, AZ 8572, USA; ^2^HRL Laboratories, LLC, Malibu, CA 90265, USA; ^3^Department of Psychology, University of Arizona, Tucson, AZ 8572, USA

## Abstract

The human hippocampus receives distinct signals via the lateral entorhinal cortex, typically associated with object features, and the medial entorhinal cortex, associated with spatial or contextual information. The existence of these distinct types of information calls for some means by which they can be managed in an appropriate way, by integrating them or keeping them separate as required to improve recognition. We hypothesize that several anatomical features of the hippocampus, including differentiation in connectivity between the superior/inferior blades of DG and the distal/proximal regions of CA3 and CA1, work together to play this information managing role. We construct a set of neural network models with these features and compare their recognition performance when given noisy or partial versions of contexts and their associated objects. We found that the anterior and posterior regions of the hippocampus naturally require different ratios of object and context input for optimal performance, due to the greater number of objects versus contexts. Additionally, we found that having separate processing regions in DG significantly aided recognition in situations where object inputs were degraded. However, split processing in both DG and CA3 resulted in performance tradeoffs, though the actual hippocampus may have ways of mitigating such losses.

## 1. Introduction

We make sense of the world by comparing our immediate sensations with memories of similar situations. A very basic type of situation is an encounter with objects in a context. For example, objects such as a salt shaker, a glass, and a sink are expected in a kitchen. Even if these objects are encountered in an office, they suggest a kitchen-like function to the area (e.g., it is a kitchenette—not a work cubicle). In other words, the objects evoke the context in which they have been experienced in the past, and the context evokes objects that have been experienced there. The hippocampus, which is essential for the storage and retrieval of memories, is likely to play a central role in this associational process. 

In rats, the hippocampus is oriented along a dorsal-ventral axis, while in primates this axis becomes an anterior-posterior axis. In both species, signals reach the hippocampus via the entorhinal cortex (EC layers II and III), which can be divided into lateral and medial portions (denoted LEC and MEC, resp.). Both the LEC and MEC can be further subdivided into caudolateral and rostromedial bands, with the caudolateral bands projecting mainly to the posterior half of the hippocampus and the rostromedial bands projecting mainly to the anterior half [1]. Within the hippocampus, these entorhinal projections reach the dentate gyrus (DG) and CA3 via the perforant path, as well as CA1. Because of the low probability of activation of its neurons, DG is thought to be responsible for producing a sparse representation of a given input which has minimal overlap with other input patterns, thereby reducing interference [2]; however the role of DG in memory is still in question [3–5]. DG projects to CA3 via the mossy fibers, a set of very strong but sparse connections. In addition to receiving inputs from DG and EC, CA3 also has many recurrent connections which are believed to serve a pattern completion purpose, allowing details lost in the sparse DG representation to be recovered in CA3 via recurrent activity and the help of EC perforant path inputs [6, 7]. The proximal region of CA3 (relative to DG) then projects to the distal portion of CA1, while the distal region of CA3 projects to the proximal portion of CA1 [8]. These connections occur in both the anterior and posterior sections of the hippocampus, with each having its own relatively independent (except in the intermediate area between anterior and posterior) DG, CA3, and CA1 subareas.

CA1 receives input from EC, with the distal portion of CA1 receiving input from LEC and proximal CA1 receiving MEC input. CA1 is essential for proper hippocampus function, since CA1 lesions result in anterograde amnesia [9]. The function of CA1 is not fully known however, although several ideas have been suggested based on theoretical [6, 7] or experimental considerations [10, 11]. We propose below a novel role for the distal and proximal areas of CA1. Each of these CA1 regions then sends output to other parts of the brain via two main pathways. The first is via the subiculum (where CA1 proximal connects to the distal part of subiculum and vice versa for CA1 distal) and to EC layers V and VI. The second pathway is via the fornix, which projects to the mammillary bodies and the thalamus.

LEC receives input mainly from perirhinal cortex and MEC receives most of its inputs from parahippocampal cortex (or postrhinal cortex in rats) which receives highly processed sensory information [12]. In this paper, we will refer to information about both the surrounding environment and spatial position within this environment, carried by the MEC, as the “context,” and the information carried by LEC as the “object,” which may include relational and configural information about objects [13]. It has been shown that in rats, MEC neurons display highly specific spatial grid fields, whereas LEC neurons have only weak spatial specificity [14]. This supports the notion that spatial environmental information arrives at the hippocampus primarily through MEC, whereas nonspatial information (what we call object information) is conveyed through LEC [10, 14]. Note that although our definition of context is based on the physical environment, other equally valid definitions are possible. For example, in a word list memorization task, context can refer either to the list in which a word appears (if there are multiple lists) or to a “processing context” that describes the actions done during the processing of the word, such as counting the number of vowels. It can also refer to a “temporal context” that describes, for example, whether a word was learned later or earlier during a session [15]. In the temporal context model (TCM) [12] and context maintenance and retrieval (CMR) framework [13], context is defined as an internally maintained pattern of activity different from the one corresponding to perception of the item itself. This context, consisting of background information about the object, changes over time and becomes associated with other coactive patterns.

The most obvious use of this incoming object and context information would be to associate and store object and context memories in hippocampus. However, while the necessity of hippocampus for spatial context recognition and navigation is well documented in rats [16, 17], various studies on the role of the rat hippocampus in object recognition have returned surprisingly mixed results. Several studies have found that novel object recognition in rats is impaired following hippocampal damage [18], temporary inactivation of the dorsal region [19], or attenuation of LEC inputs to the dorsal region [10]. These experimental results suggest that detailed information about the world may indeed be represented within the dorsal hippocampus and may be dissociable from contexts, while other studies have concluded that only contextual information is stored in hippocampus [20, 21], or that the hippocampus is not required for intact spontaneous object recognition memory [22]. Analysis of neural spike data during an object recognition memory task in rats showed that hippocampal pyramidal cells primarily encode information about object location but also encode object identity as a secondary dimension [23]. Manns suggested that objects were represented mainly as points of interest on the hippocampal cognitive map, and that this map might aid the rat in recognizing encounters with particular objects [23]. 

In humans, the question of where memory for objects is stored is still debated, although patients such as H.M. and K.C. who have had bilateral hippocampus removals demonstrate that the hippocampus is required for the formation of new object memories and recall of most short- and medium-term memories (those formed within the last several years) [24, 25]. It is known that the human hippocampus is active during object-type recall [26]. Specifically, during successful memorization of word lists, there is significantly more activation of the posterior hippocampus than the anterior hippocampus [27]. A greater degree of posterior activation is also seen during the encoding of novel pictures [28]. However, the posterior region often responds to spatial tasks as well, particularly those concerning local spatial detail (see [29] for a review of differences in spatial and other types of processing between the anterior and posterior regions). In this study we assume that both specific object and context representations exist and are stored as memories within the hippocampus. While both regions seem to process spatial contextual information, only the posterior region has been strongly implicated in object memory as well. We therefore hypothesize that the anterior region of the primate hippocampus is primarily processing contextual information, while the posterior region is relatively more object oriented. The models that we develop in this study have explicit object recognition as a main feature and should therefore mainly be considered models of the primate hippocampus because of the evidence for explicit object representations in this case. We will discuss how our models can be related to the rat hippocampus in Section 4.

In summary, we assume that object and context memory are mainly stored in the posterior and anterior regions of hippocampus, respectively. Recall, however, that the posterior region also receives input from the caudolateral band of the MEC (which carries contextual information), and the anterior region receives input from the rostromedial band of the LEC (which carries object information). These connections raise the question of the purpose of having both object and context information reach the posterior and anterior subdivisions of the hippocampus. Recent reconsolidation experiments have shown that spatial contextual information plays a significant role in object retrieval and encoding [30, 31]. We propose that the MEC connections to the posterior stream mentioned above are vital for this. The experiments we describe next explain why context plays such a pivotal role in memory. We provide evidence that elements of hippocampal anatomy such as differentiation between the blades of DG and functional separation of the distal and proximal regions of CA1 may work together to improve the selective use of context information in object recognition, and that this can in turn improve memory performance in certain situations.

Overall, we attempt to formulate a coherent explanation for the role of several distinct anatomical features of the hippocampus and how they work together. This explanation centers on the idea that some of these anatomical differences may have evolved in order to deal with the two intrinsically different types of information that enter the hippocampus through LEC and MEC. These two types of information are “object” information (specific items within an environment, e.g., a spoon) and contextual information (the environment itself—generally less numerous than objects and related to general classes of objects, e.g., the kitchen).

Our hypothesis is that the anatomical features of the hippocampus can help manage the flow of these two types of information better than an undifferentiated hippocampus could—that they allow these two types of information to come together only in areas where it is beneficial and keep them apart otherwise. The question we are addressing in this paper is the following: can these anatomical features actually improve performance by playing the information managing role that we have proposed? We determine this by testing on a number of basic memorization tasks and find that the models with these features do indeed perform better than the baseline model on some of the tasks.

Why would we want to examine this question? There has been a large amount of work done on the theoretical aspects of how the hippocampus stores generic inputs and what role each of the main subregions (DG, CA3, and CA1) may play. In recent years, however, anatomical studies have demonstrated that there is a high degree of differentiation in terms of connectivity along multiple axes of the hippocampus (posterior-anterior and distal-proximal) and within each of the subregions. At the same time, experimental studies have shown that this differentiation has actual consequences for the memorization ability of different regions, and the studies above have shown that context plays an important role in object memorization. Thus, it is important to consider how these new findings fit into the theoretical picture of how the hippocampus works. We can no longer just consider the hippocampus or its subregions as single blocks (CA1, CA3,…) nor consider all inputs as homogeneous if we are to have any hope of explaining existing behavioral data at the neural network level. We come at the question of how the anatomical data can explain the new experimental data with two important ideas that we believe have not been adequately expressed up to now: (1) that the anatomical features mentioned above play an information managing role whose existence only becomes necessary once we start to consider at least two different types of information converging in the hippocampus and (2) that the roles of these individual features only make sense when looking at their interaction with everything else; for example, differentiation within DG on its own would be less useful for managing information if the rest of the upstream regions like CA1 did not also have features (like the proximal-distal distinction in our model) that make use of how DG partitions this information. 

## 2. Methods

### 2.1. Model Structure and Connectivity

We use an expanded version of a model of the hippocampus developed by O'Reilly et al. [32]. The original model is a basic hippocampus consisting of a single input (EC layers II and III), a DG, CA3, and CA1 layer and a single output (EC layers V and VI). This model includes recursive connections within CA3 and DG to CA3 connections that are 10 times stronger than the EC to CA3 connections to mimic the sparse but powerful mossy fiber synapses. The smallest computational element is a “unit,” which simulates a small population of neurons in a rate-coded fashion [33]. We will use the term neuron synonymously with unit in the rest of the paper. The network is trained using the Leabra algorithm, which is based on the generalized recirculation algorithm. Unlike the original model, we do not pretrain the EC → CA1 → output connection. In addition, we did not model an explicit EC output layer; we simply have an output layer. Further details of the original model can be found elsewhere [6, 34].

**Figure 1:**
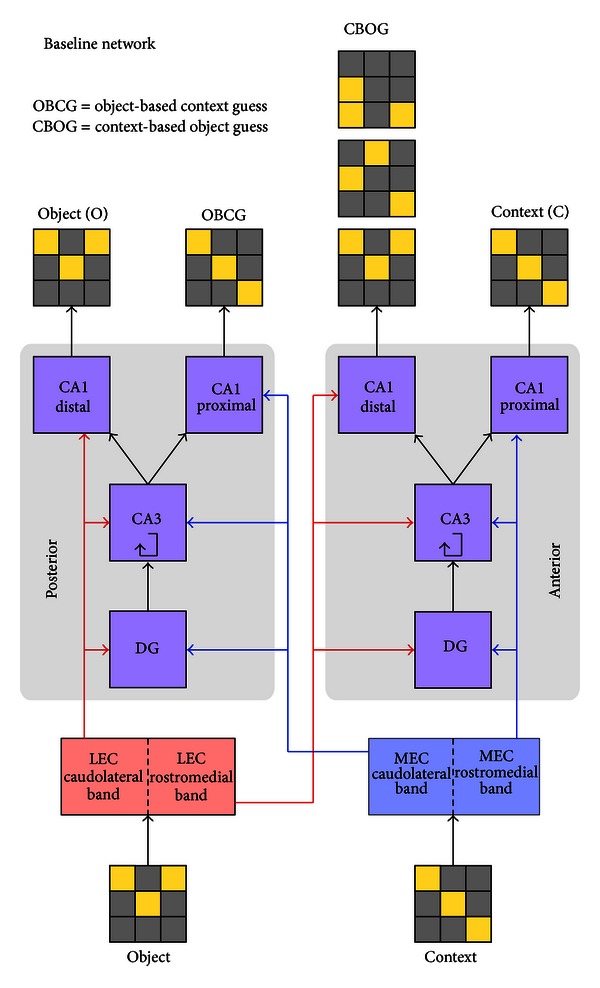
Layer and connectivity diagram of the Baseline network. Matrices representing an object and a context are the inputs to the network. The outputs are an object (O), an object-based context guess (OBCG), a context-based object guess (CBOG), and a context (C). The OBCG output is the context that the input object is associated with during training, and the CBOG output is the set of objects that were associated with the input context during training.

Our model explicitly separates the posterior and anterior halves of the hippocampus, so that the network has two CA3 regions, two DG regions, and two CA1 regions, each in the posterior and anterior poles. EC is split into lateral and medial regions (LEC and MEC, resp.), with LEC connected to all three layers on both the posterior and anterior sides to simulate the outputs of the caudolateral and rostromedial bands, respectively, and similarly for MEC. As supported by the neuroanatomy, CA3 proximal (in relation to DG) connects to CA1 distal and CA3 distal connects to CA1 proximal [8]. In order to model this distal/proximal connectivity distinction, we split each of the two CA1 regions into half again, to give four separate CA1 regions (two on the posterior side and two on the anterior side). Each CA1 receives input from the ipsilateral CA3 along with either LEC input (if it is distal) or MEC input (if it is proximal). This network will be referred to as the “Baseline” network (Figure 1).

We model inhibition in each layer as a competitive k-winner-take-all process, where only the top k most active neurons send their outputs to the next layer. Thus we can set the activity level in each region to approximately that seen in experimental results, where the activity level refers to the percentage of active neurons at any given time. EC, DG, CA3, and CA1 have experimental activity levels of 7%, 1%, 2.5%, and 2.5%, respectively [34]. In our model, these levels are set to 25%, 1.5%, 2.3%, and 2.5%, respectively. The discrepancy in EC (both LEC and MEC) is because it is serving as our input layer and does no computation; EC is just large enough to hold training patterns with 25% of the units active. The LEC and MEC layers each consist of 64 neurons. The DG, CA3, and CA1 layers on the posterior side consist of 800, 256, and 800 neurons, respectively (the distal and proximal regions of CA1 have 400 neurons each). The same numbers apply on the anterior side.

As discussed above, the LEC primarily carries object information while the MEC carries spatial contextual information. Hence in our model we conceptualize the LEC inputs as “objects” and MEC inputs as “context.” In assigning roles to the output layers corresponding to the distal and proximal CA1 regions, we first note that these two regions lie on largely separate output pathways: CA3 proximal connects mainly to CA1 distal and CA1 distal connects mainly to the proximal part of the subiculum, which in turn projects back to the LEC [8, 35]. On the other hand, CA3 distal connects mainly to CA1 proximal and CA1 proximal connects to the distal part of the subiculum, which in turn projects back to the MEC [8, 35]. If these pathways were both carrying the same type of information, there would be no need for such a wiring scheme to keep them separate. Since our model only contains two types of information, object and context, we assume that one of these pathways is carrying object information and the other is carrying context. 

On the posterior side of hippocampus we are mainly focused on its object processing capabilities; hence we assume that the relevant outputs must be largely dependent on using object-type information from LEC. We hypothesize that these two outputs are an object guess and an object-based context guess. The object guess pathway does standard object recognition by taking the input object, matching it to the closest object in memory, and giving the best match as its output. The object-based context guess pathway uses the object input to generate the context that the object is associated with: if one gives it the object “swing set,” it returns “playground,” if one gives it “refrigerator,” it returns “kitchen,” and so forth. We emphasize that not every neuron in the given regions is doing these operations or using only one type of information to do them. But, to the extent that we have neurons that are encoding nonspatial information in these regions, we predict that there will be more of them (or alternatively, that the degree to which they are sensitive to spatial information will be lower) in the distal region of CA1 compared to the proximal region. Experimental results by Henriksen et al. provide support for this, showing that the strongest spatial modulation occurs in the proximal part of CA1, and that distal CA1 cells are less spatially tuned [36].

On the anterior side of the hippocampus, since we focus on its contextual processing capabilities, we require that its outputs be largely dependent on using context-type information from MEC. We hypothesize that these two outputs are a context guess and a context-based object guess. The context guess pathway matches the input context to the closest context in memory, and the context-based object guess uses the input context to generate a list of the set of objects associated with the given context. For example, given the context input “playground,” it would output the object list “swings, sand-box, slide.”

The final question is which of the distal or proximal CA1 regions is playing each of these roles. It is known that MEC projects preferentially to the proximal region of CA1, while LEC projects preferentially to the distal region [37]. Assuming that the purpose of the two CA1 streams is to keep object and context-type information largely separate, it seems unlikely that object information from LEC would then be projected to the context stream at CA1, and similarly for MEC inputs and the object stream. Thus, on the posterior side, we conclude that the object guess is output by distal CA1 and the object-based context guess is output by proximal CA1. Similarly, on the anterior side, we conclude that the context-based object guess is output by distal CA1, and the context guess is output by proximal CA1.

### 2.2. Model Variants

**Figure 2:**
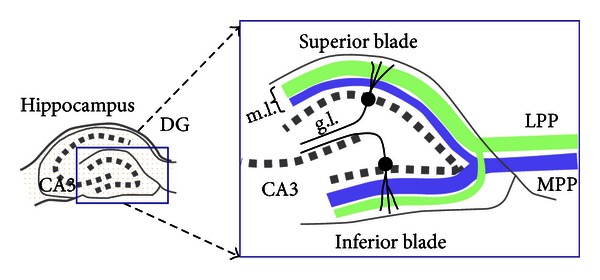
Connectivity of lateral perforant path (LPP) and medial perforant path (MPP) inputs to superior and inferior blade of DG. The LPP and MPP fiber lamina are thicker on the superior blade and inferior blades, respectively, resulting in higher effective synaptic weights (adapted from [38]).

Variants of the Baseline network were designed to investigate the effect of two additional anatomical details. The first is the differentiation between the inferior and superior blades of DG. As shown in Figure 2, the DG may be functionally separated into two parts because of the different strengths of LEC and MEC connections onto the superior and inferior blades and a postulated dendritic gating mechanism [38, 39]. Both blades receive proximal dendritic MEC input via the medial perforant path (MPP) and distal dendritic LEC input via the lateral perforant path (LPP). However, the superior blade receives stronger LPP input whereas the inferior blade receives stronger MPP input. We further hypothesize that the effect of this connectivity is different depending on whether the given DG region lies in the posterior or anterior hippocampus. 

In the posterior hippocampus, the object information contained in the LPP input is more relevant to its task than the context information coming from the MPP input. Thus we would expect that the DG neurons in posterior hippocampus would be biased toward (or learn to weight more heavily) the LPP inputs over the MPP inputs. However, the fact remains that the MPP inputs are more proximal to the soma and thus cannot be completely ignored. The hypothesized result of this tug-of-war (more relevant LPP input but more proximal MPP input) is that, in the superior blade where the LPP object inputs are already stronger than the MPP context inputs, LPP is able to largely control the neurons' firing. In the inferior blade where LPP inputs are weaker, they are able to achieve approximate parity with the MPP input. 

In anterior hippocampus the MPP contextual inputs are both more relevant and more proximal to the soma. We hypothesize that this allows the MPP inputs to control the neurons' firing, though to a greater extent in the inferior blade than the superior blade, where LPP input cannot be totally ignored. 

**Figure 3:**
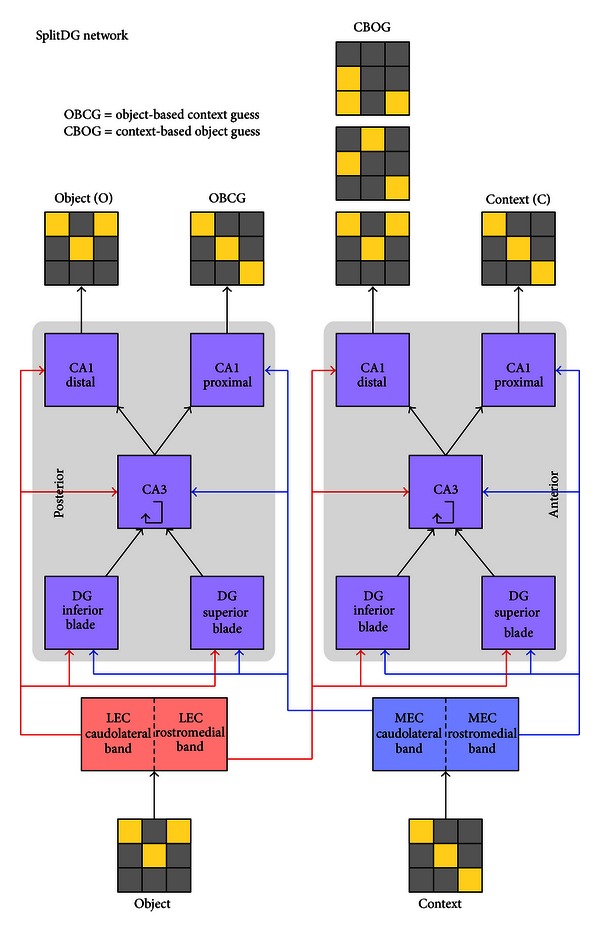
Layer and connectivity diagram of the SplitDG network.

 We model the two blades of DG as separate layers in both the anterior and posterior sides of hippocampus in order to determine their effect on performance. The model with DG layers split in this way, but with all other architecture the same as in the Baseline model, will be referred to as the “SplitDG” model (Figure 3).

**Figure 4:**
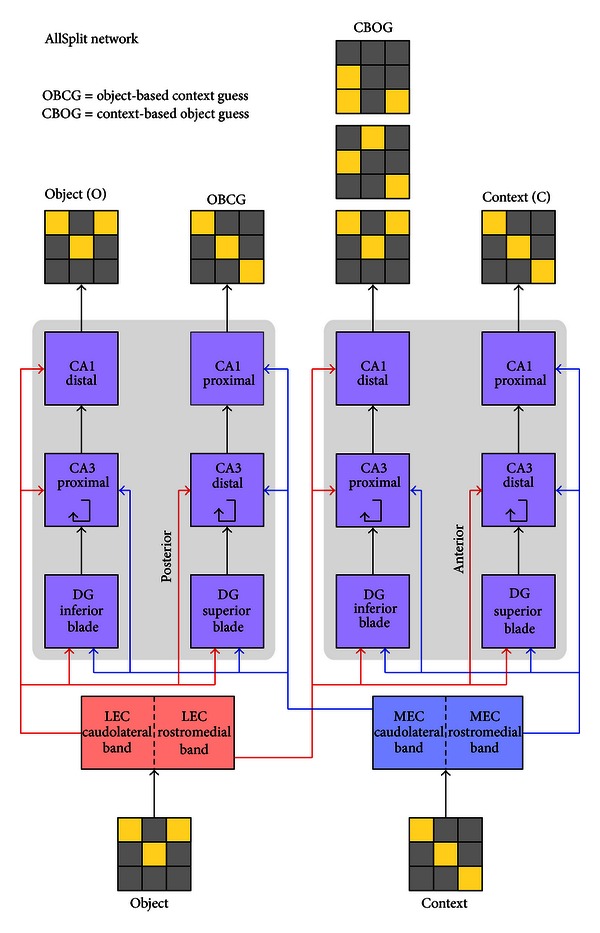
Layer and connectivity diagram of the AllSplit network.

The second anatomical detail we consider is differentiation between the proximal and distal regions of CA3. As mentioned in the introduction, CA3 has distal and proximal regions just as in CA1 (here distal and proximal refer to distance from DG, rather than to the location on the dendrite). These regions receive different amounts of inferior and superior blade DG input and have distinct patterns of recurrent connections [8]. The amount of recurrent versus feed-forward connections is also different between the two subareas. Thus these two regions of CA3 may be performing functionally different roles. In order to determine the purpose of such a split and test whether it may confer some performance advantage, we construct a third network that has CA3 split into two layers on each of the posterior and anterior sides, in addition to the DG split described above. Anatomically, the inferior blade of DG projects to proximal CA3, while the superior blade projects to both proximal and distal portions of CA3 [8]. As a modeling approximation we connect the inferior blade to proximal CA3 and the superior blade to distal CA3 only. Although our model does not capture the detailed connectivity of CA3, we believe it serves as a good starting point for understanding the purpose of having distinct CA3 regions. We will refer to this network as the “AllSplit” network (Figure 4).

### 2.3. The “+” Networks

We constructed two additional networks, SplitDG+ and AllSplit+, for the purposes of comparison across networks with equal training set error. SplitDG+ is the same as SplitDG, except that each of the DG layers is doubled in size. Similarly, AllSplit+ is the same as AllSplit, except that both the CA3 and DG layers have been doubled in size. The relevance of these networks is addressed in more detail in the discussion.

### 2.4. Training and Test Sets

**Figure 5:**
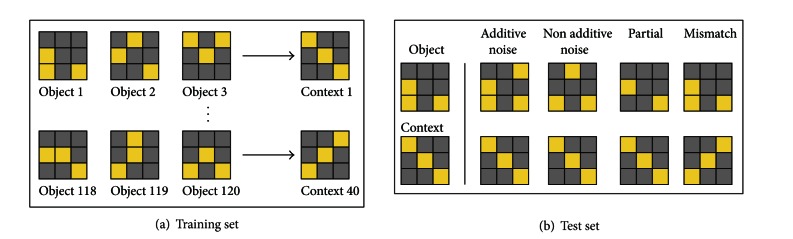
Training and test sets. The training set consists of 120 objects and 40 contexts, with 3 objects per context. The test sets are the same as the training set, except with either noise added (additive or nonadditive noise), part of the pattern missing (partial cue), or an object and context mismatch.

The training set consists of object patterns and context patterns (Figure 5). Each object is a random 8 × 8 matrix of zeros and ones, consisting of 16 ones (active units) and 48 zeros (inactive units). Contexts are constructed the same way. There are 120 unique objects and 40 unique contexts (3 unique objects per context).

The output layers of the network are referred to as “object” (O), “object-based context guess” (OBCG), “context-based object guess” (CBOG), and “context” (C). The correct output for the object output layer (used as a training signal and ground truth for the error metric) is the object matrix for the input object. For the OBCG layer, the correct output is the context matrix associated with the given object input. For the CBOG layer, the correct output is the three object matrices for the three objects associated with the given context. Finally, for the context output layer, the correct output is the context matrix for the input context.

The network is trained for 20 epochs, where each epoch consists of presenting all 120 object-context pairs in a random order and applying the Leabra weight update algorithm after each presentation. Twenty epochs were chosen as the stopping point because all networks' training error had stabilized at close to their minimum value by this time. 

After training, the networks' weights are frozen, and the networks' performance is measured using four test sets: additive noise, nonadditive noise, partial cue, and context mismatch (Figure 5). In additive noise tests, objects or contexts have some of the zeros in their matrix replaced by ones, simulating additional active units. In non-additive noise tests, for each zero that is replaced by a one, a one from the original pattern is replaced by a zero, so that the total number of active units remains the same. In partial cue tests, some of the ones in the original object or context pattern are replaced by zeros, resulting in a fewer number of active units overall. In the context mismatch test, an object is paired with a different context from the one it was associated with during training. The level of difficulty of each test depends on the number of units that are changed from the original pattern, which we denote by percentages in the figures.

Many experimental or real-life situations can be interpreted in terms of these simple tests or a combination of them. For example, if the object we are memorizing is a man's face, we recognize who he is even if he has grown a mustache (additive noise), is wearing a hat (non-additive noise, since it adds something but also covers his hair, which is one of his original features), or is partially turned away from us (partial cue). In addition, we recognize him even if we see the same man in a different context (mismatch), although this may be a somewhat more subtle issue than the previous ones, which we will discuss further.

## 3. Results

### 3.1. Setting the Crossconnection Weights for the Baseline Model

**Figure 6:**
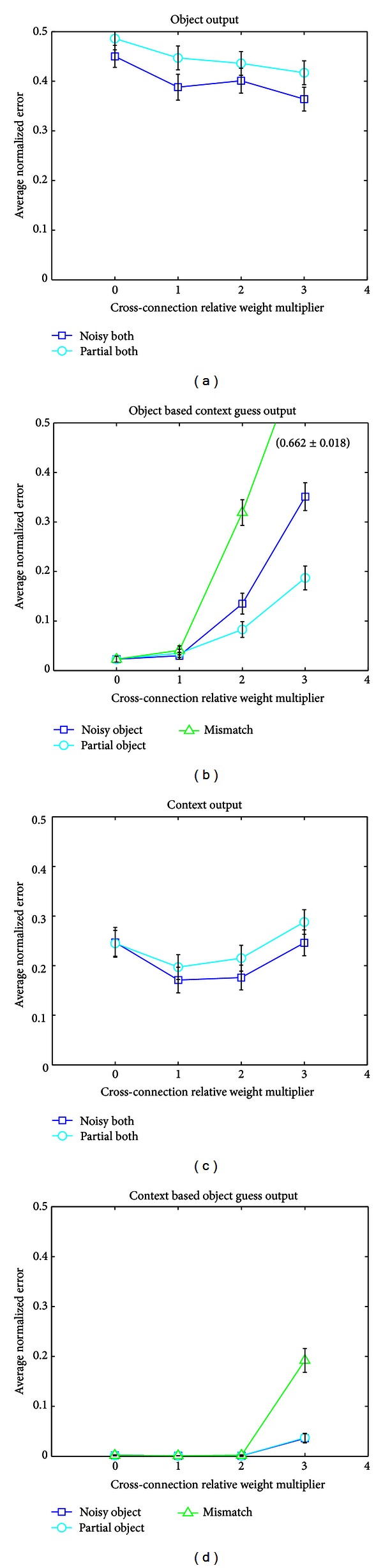
Error for each of the four output layers of the Baseline network on various sample tasks, as a function of crossconnection weighting. Crossconnection input refers to LEC input to anterior hippocampus and MEC input to posterior hippocampus. Higher relative weight multiplier values mean stronger MEC input to posterior and stronger LEC input to anterior streams. (a) Object output error on noisy and partial cue tests (where both object and context are noisy or partial, resp.) as a function of crossconnection strength. (b) OBCG output error on noisy and partial cue tests (here the noise and partial are only in the context) as a function of crossconnection strength. (c) Same as A, except the error is measured at the context output layer. (d) Same as B, except only the object is noisy or partial, and the error is measured at the CBOG output layer. Error bars are standard errors of the mean.

We will refer to the connections from LEC to the anterior side of hippocampus and from MEC to the posterior side as “crossconnections,” since they bring object information into the context-dominated anterior side and context information into the object-dominated posterior side, respectively. The first task was to determine how the relative amount of crossconnection and noncrossconnection input affects the error rate of the Baseline network and use this to maximize its performance. Since the OBCG and CBOG output layers are used in different situations from the O and C layers, we test them accordingly on a different set of tasks. The O and C layers were tested on a set with mixed additive and non-additive noise introduced to object and context (15% noise in each layer) and a set where both object and context were incomplete (40% complete each). The OBCG layers were tested when object and context were mismatched, with noise (30%) in context only, and partial (40%) in context only. For the CBOG layer, the mismatch test was the same, but the noise and partial tests were in the object input only (30% object noise and 40% partial object) rather the context. The results can be seen in Figure 6. 

**Figure 7:**
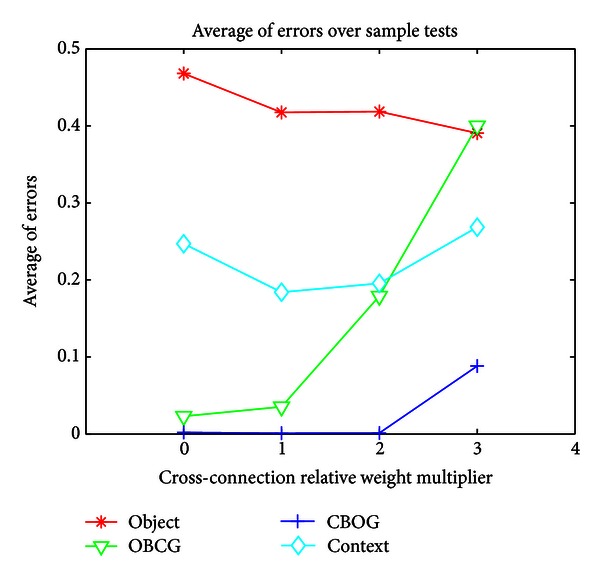
Average of errors over sample tests for each output layer, as a function of crossconnection strength.

To determine the optimal LEC and MEC weights for each output stream, we plot each output layer's average error over the set of relevant tests as a function of the crossconnection input it receives. This is shown in Figure 7. We use this as a guide to set the relative weights of the crossconnections for all the networks to levels which optimize their performance on the sample tests. Note that for networks such as SplitDG or AllSplit which have split layers, we optimize the crossconnection strengths for these layers independently, while for the Baseline network, we must average the optimal connection strengths over the two output types. For example, since the O output does best with a multiplier of 3 while OBCG does best with a multiplier of 0, we end up with the Baseline network having a relative weight multiplier of 1.5 for the MEC to dorsal side crossconnections. For the AllSplit network, we do not need to make this compromise and can directly use a multiplier of 3 for the MEC inputs into the DG and CA3 areas which feed into O and use a small multiplier close to 0 for the DG and CA3 areas which feed into OBCG. The SplitDG network has the same weighting for crossconnections to DG and CA1 as the AllSplit network and the same weighting to CA3 as the Baseline network, since it only has a single CA3 which the O and OBCG streams must share. These results show that there is unlikely to be a single set of crossconnection weights that optimizes performance for the various output layers across a range of different tasks. The flexibility provided by having different DG and CA3 layers that can take different levels of crossconnection input provides an advantage and may be one of the reasons why this anatomical differentiation exists in the hippocampus.

### 3.2. Training Error

**Figure 8:**
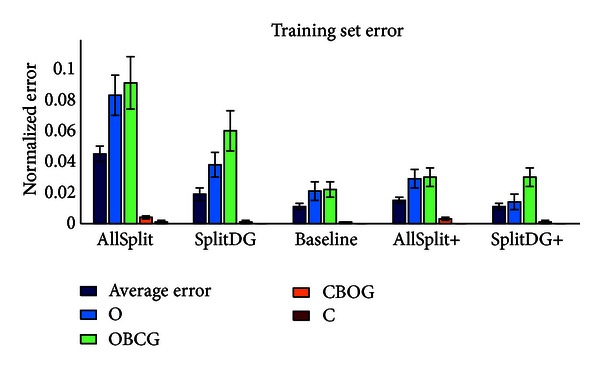
Error on the training set for each of the five networks after 20 epochs of training.

Having fixed the crossconnection weights in all networks to values that minimize the error over the sample test sets, we now compare the networks. First we measure the error on the training set after 20 epochs, when the error has reached its asymptotic minimum.  Figure 8 shows the average error for each of the five networks, along with the error on each of the four outputs individually. The networks can be divided into two categories for further comparison: those which have the same number of neurons, consisting of AllSplit, SplitDG, and Baseline and those which have the same initial training set error, consisting of Baseline, AllSplit+, and SplitDG+. This illustrates the fact that differences in layer size may play an important role in the networks' basic memorization ability. When a layer is split, each of the halves can specialize more efficiently on the task, for example, pattern completing an object or converting an object to a context guess. On the other hand, it must hold the same number of object or context memories despite being half the size, resulting in more memorization errors.  Figure 8 shows two possible outcomes of this tradeoff: for the context and CBOG streams, there is no difference in training error before and after splitting the CA3 and DG layers which lie on those streams (compare C and CBOG error between Baseline, SplitDG, and AllSplit). This is due to the fact that these layers only need to store 40 context memories, so even when they are split in half they have no difficulty memorizing them all. However, for the object and OBCG streams, splitting their respective DG or CA3 layers results in a significant increase in training error (compare O and OBCG error between the same three networks). In this case they need to memorize 120 objects, and a CA3 or DG layer half the size is not sufficient. The results of the “+” networks show that this is no longer a problem if we simply have more neurons to start with. The question of whether it is more appropriate to compare Baseline with AllSplit+ and SplitDG+ (since they start off with the same training set error) or to compare Baseline with AllSplit and SplitDG (since they have the same number of neurons) depends on which situation is more likely to reflect biological reality and will be addressed further in the discussion. In all subsequent tests we include the results for each of the five networks.

### 3.3. Test Sets

We seek to determine how, and in what situations, contextual information can be used by the hippocampus to aid in object recognition and recall (and similarly how object information can aid context recognition), and what role differentiation within DG and CA3 may play in using this information. To answer these questions, we have constructed three primary networks with varying degrees of differentiation in the DG and CA3 layers and will test the ability of each of these networks to recognize objects and contexts under various conditions of degraded inputs. 

**Figure 9:**
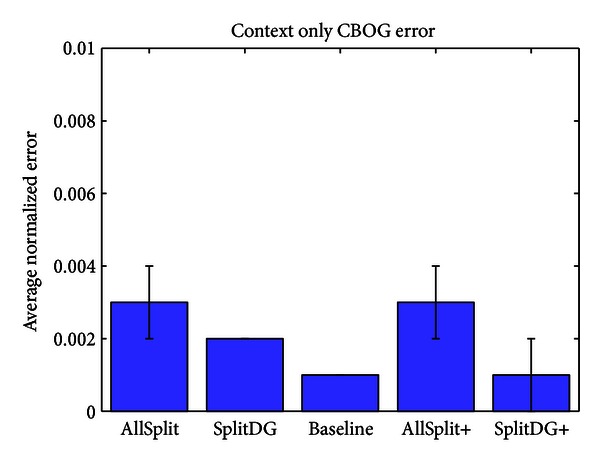
Error on the context-based object guess (CBOG) output when given only the context as input.

A common and simple test of human memory is to have a subject memorize a list of words or set of objects, then recall them given a cue. We would like to determine if our network is capable of giving this object output even without the object input. We simulate this task in our networks by presenting a context (the cue—which would consist of the room and the experimenter) and use the CBOG output to get a list of the objects which have been memorized in the given context.  Figure 9 shows that the CBOG stream performs well in this task. There is little difference between networks here since all use the same crossconnection strength into the anterior side, where CBOG is located.

**Figure 10:**
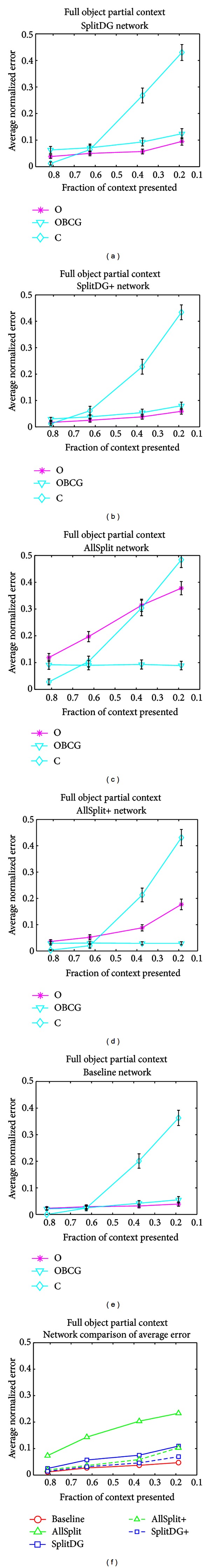
Error for each of the networks' O, C, and OBCG layers when a partial context and full object were given as input. (a) SplitDG, (b) SplitDG+, (c) AllSplit, (d) AllSplit+, (e) Baseline, and (f) average error across the object output and the lowest of the two context outputs (C or OBCG) for each network, as a function of percentage of context input presented.

Next we consider the case where the context, rather than the object, is missing to various degrees. This test will help us determine the degree to which relying on contextual input to recognize objects is disadvantageous when the context is degraded.  Figure 10 shows the individual performance of the output layers O, OBCG, and C as a function of how much of the context is given for the various networks, illustrating the effect of having increased MEC inputs into the object stream. Because the AllSplit network's object stream uses a relatively large amount of context information, partial context input has a greater adverse effect on the AllSplit network's O output than it does on the Baseline network's O output. The same is true for SplitDG and its “+” counterpart. Thus we do not expect the AllSplit network to do well compared to the Baseline network in this situation, and Figure 10(e), which gives the average error for each network by taking the average of the error from the O output and the best context output (either C or OBCG), confirms this. The “+” networks do relatively better since their larger CA3 sizes allow the partial context-object mix within the object stream to be pattern completed to a higher degree. This figure also shows the advantage (for all the networks) of having an OBCG output when context is difficult to discern. When the fraction of context drops below 60%, the networks can rely on OBCG for their context guess rather than the context stream output C.

**Figure 11:**
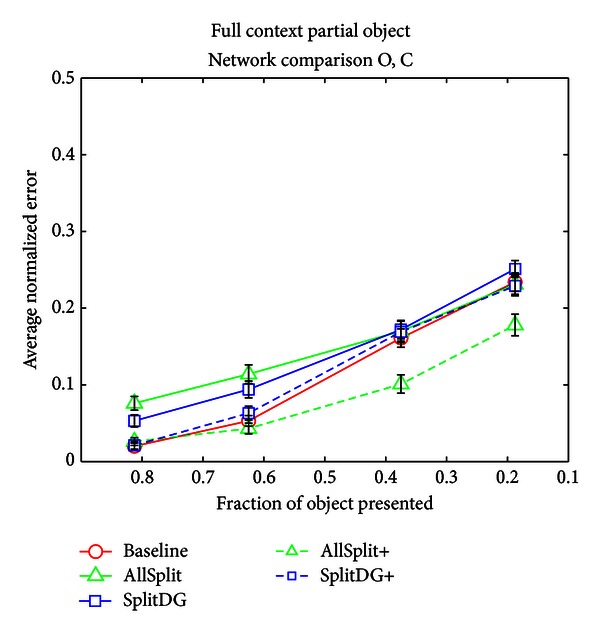
Average error on the O and C output layers when full contexts and partial objects were given as input.

The analogous situation on the object side is to present a partial object and a full context. This test helps us determine how well the various network architectures can utilize context to aid object recognition. At first glance it seems that we ought to make use of the CBOG output to generate an object guess using the clean context, just as we used the OBCG layer in the partial context case above. However, the problem is that the CBOG layer activates multiple possible objects rather than a single object, and thus we would need a way of picking the correct object out of this list. Cortical areas outside of hippocampus could conceivably accomplish this by picking the closest match either to the original input or the O output; however, since we restrict our model to the hippocampus proper, we have not attempted to implement such a scheme and instead use the O output as our exclusive object guess. We consider this issue further in the discussion.  Figure 11 shows that, when the object is partially given, the increased amount of context information that the AllSplit network uses via the MEC to posterior crossconnections becomes an advantage rather than a liability, as it now has an error rate similar to that of the Baseline network. When the initial training set memorization disadvantage is accounted for under the AllSplit+ network, a consistent advantage for all partial conditions is seen. Surprisingly, neither SplitDG nor SplitDG+ is able to do better than the Baseline network, suggesting that some degree of heterogeneity within CA3 is necessary to take advantage of the additional context information.

**Figure 12:**
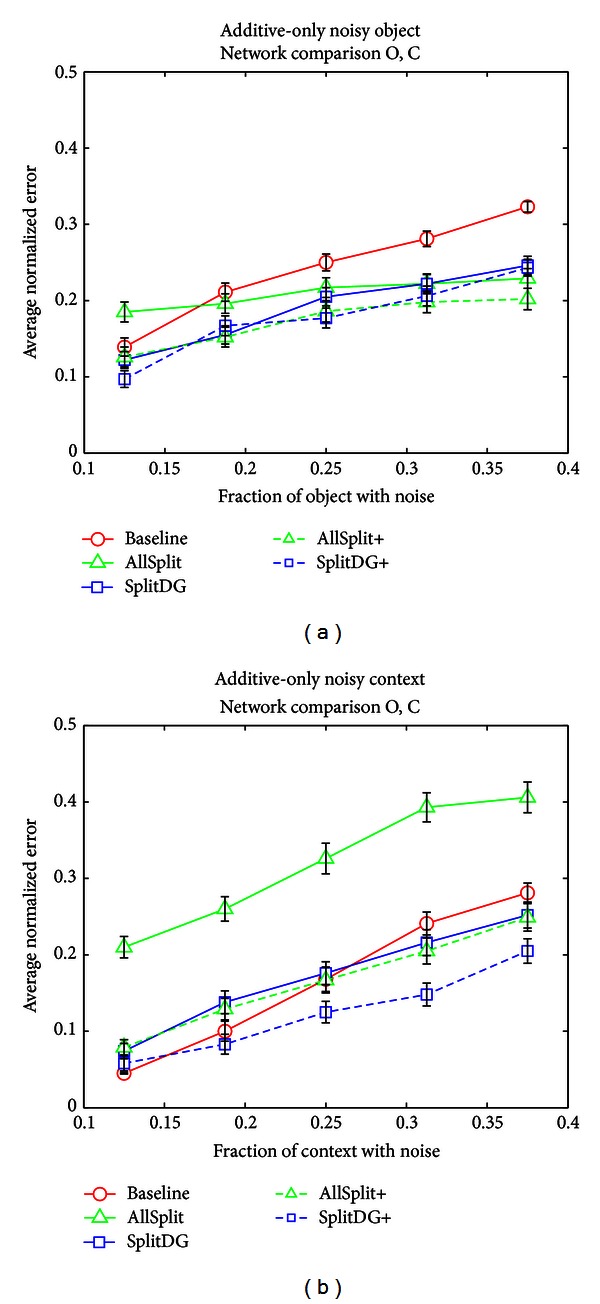
Additive-only noise tests. (a) Error across networks, averaged over the O and C output layers, when noisy objects and noiseless contexts were presented as input. (b) Error across networks, averaged over the O and C output layers, when noiseless objects and noisy contexts were presented as input.

Figure 12 illustrates the effect of having additive-only noise in the object or context input layers. These tests are of the same nature as the partial input tests done previously and are designed to determine if there is any difference in how the networks deal with noise, and whether this allows more or less effective use of the crossconnection inputs. As with the partial object case, the AllSplit network performs well with object noise by using the additional context information available to its object stream to help it guess the object. In this case, the SplitDG and SplitDG+ networks also do better than the Baseline network and about the same as their AllSplit counterparts, though slightly worse in high noise situations. When the noise is in the context input, AllSplit does worse since it must deal with additional noise in its object representation. The larger DG and CA3 areas of the SplitDG and “+” networks clearly help with this task and bring performance on par with or even better than the Baseline network (in the case of SplitDG+), indicating that even if the context input is highly noisy, a large CA3 can extract enough additional context information to aid in object identification.

**Figure 13:**
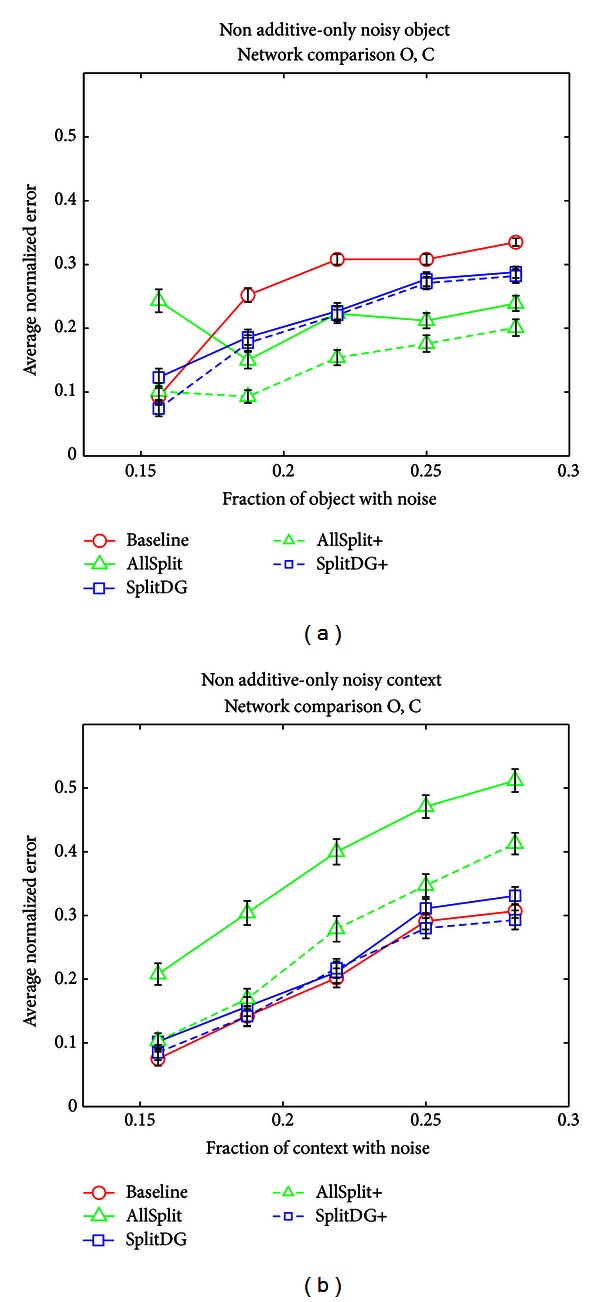
Nonadditive noise tests. (a) Error across networks, averaged over the O and C output layers, when noisy objects and noiseless contexts were presented as input. (b) Error across networks, averaged over the O and C output layers, when noiseless objects and noisy contexts were presented as input.

Figure 13 shows the results of the non-additive noise task. As in the additive-only task, the split networks perform better than the Baseline network when the object is noisy, with the AllSplit network performing better than SplitDG. When the context is noisy, the pattern is reversed, although SplitDG does just as well as the Baseline network.

## 4. Discussion

### 4.1. Anterior-Posterior Crossconnections

The results in Figure 6 suggest that a split network provides performance advantages compared to the Baseline network. Each output layer requires a different object to context input ratio in order to perform optimally on the relevant tasks. The object output layer gives the network's best guess as to what the actual object is, meaning it needs to perform well in low to medium noise and partial situations where either the object or context input (or both) is degraded. Surprisingly, additional contextual information is helpful even when that context is as noisy/incomplete as the object. This can be thought of as providing a “bigger picture” for the network to look at, and thus making it more likely that it can find some relevant clue which it can use to decipher the entire input. For example, suppose one is looking at a photograph of a person taken from a side angle so it is difficult to determine who it is (partial cue). If a wider-angle photo is now given which includes some of the person's body or clothing (partial context), this information gives a clue as to who the person is, even if the full context is unavailable. The same idea applies to noisy objects and contexts. 

However, since each context contains several possible objects, the context input gives less information than the object input, and therefore its value (as far as the object output is concerned) decreases rapidly to zero with the amount of signal degradation. It is not a case when more information is beneficial regardless of how noisy it is. At some point, the error introduced by the noise outweighs the value of having additional information. If the object is presented noiselessly, then additional contextual information is not very useful, particularly if it itself contains noise. For the CA3 size used in our AllSplit network, this point of zero benefit occurs approximately when the context begins to have more noise or be more incomplete than the object. This is why, in the “partial context” and “noisy context” tests, we see the AllSplit network perform rather poorly with its relatively large amount of context input into the object stream (via the strong MEC connection). As we would expect, the more degraded the input context compared to the input object, the worse the AllSplit network performance. On the other hand, when the input context is less degraded than the input object, as in the “partial object” and “noisy object” tests, the AllSplit performance increases above that of the other networks. Again, because the context inputs have less absolute predictive value than the object inputs (for the object output layer) to begin with, the beneficial effect of noiseless context is less than the detrimental effect of degraded context, and as a result the noiseless context benefit does not come into play until object noise/partial levels are slightly higher. However, the beneficial effects can clearly be seen at moderate object noise levels, and for low noise levels the error is near the training threshold.

In all the networks, in the case of the context being particularly noisy/incomplete, the context output from the anterior stream may be too noisy for use. The hippocampal network would then turn to the object-based context guess output to deliver a context prediction, provided that the object input is relatively noiseless. Thus the OBCG layer needs to be effective in noisy/partial context and mismatch situations, which is what we test in Figure 6(b). In order to achieve good performance, the output must not use the MEC context input, since this layer will only be called on when the context is particularly noisy or incomplete. In addition, if the output relies too much on context, it begins to duplicate the functionality of the anterior context stream. Fortunately for the AllSplit network, this highly degraded or mismatched context situation in which C must be substituted with OBCG is also exactly the situation in which the object output fails; hence it may be able to conveniently rely on the OBCG layer's output to give it a reliable context to use. We have not implemented this backup functionality in our network. 

The context output layer is similar to the object output layer in that it must be able to deal with noise in both object and context, and dealing with object noise is of higher priority (as it is with the object output) because the OBCG layer provides a backup in the case of high context noise. For the context layer, this means that it should have a small amount of object input relative to context input.  Figure 6(c) shows that this naturally occurs thanks to the fact that there are much fewer contexts than objects, and thus the context stream is very effective at determining context even when they are noisy/incomplete. As a result additional object information is of little use to it, so the LEC to context stream input has less influence than the MEC to object stream input.

As with the context stream, the CBOG stream has fewer input-output associations to store; hence it relies less on the object input from LEC crossconnections. It is important that it depends mostly on context for the same reason that OBCG depends mostly on object, although the CBOG list may get called on even when the object input is usable, since it provides additional information that the object output cannot give. This layer provides a mechanism by which a list of objects can be recalled given only a single contextual cue. Networks consisting of only a single object and context output would not be able to model this task. One artificial feature of this output is that it is N times as large as the object output, where N is the number of objects per context (here 3). We are not implying that in the actual hippocampus, the region that distal CA1 on the anterior side projects to is N times as large or N times as active as the regions all the other CA1 areas project to. In the actual hippocampus these object outputs may come out one at a time, as the network activity has a time component in spiking networks. Since our model is strictly a rate-based connectionist model, the only way we can represent this output is as a single matrix in which all objects are represented at once. The OBCG output could also be represented this way, in the case where objects are allowed to appear in more than one context. 

The temporal dynamics of context-based object retrieval in free recall situations have been given a theoretical foundation in the TCM (temporal context model) and CMR (context maintenance and retrieval) frameworks [40, 41]. Our model explicitly represents the biological structures and connections that make possible the basic multiple object to context associations (referred to as source clustering) assumed by these frameworks, but we do not attempt to provide a realization of any of the temporal aspects of memory (temporal clustering) which TCM and its generalizations also deal with, such as associations between successively presented contexts and the recency effect. However, allowing objects to be associated with more than one context (as they are in the case of the temporal context), our model could conceivably provide a starting point for a biological realization of the TCM framework. The varying internal context of TCM could be produced within our model by having objects output by CBOG feed back into the OBCG stream to produce an associated set of contexts, which would then be used as inputs into the CBOG stream to produce the next object to be recalled, in a repeated cycle.

### 4.2. Effects of Layer Size

There are two ways to approach the interpretation of the other test results, beginning with the training set error. The first way is to ignore the size of the network and compare only those networks that have similar amounts of error on the training set. In this view, a fair comparison would be between those networks that start out with equal amounts of knowledge on the training set, regardless of how many epochs it took them to get their error to that level or how many neurons they have. Here, splitting a layer into two separate sublayers has little to no disadvantage, because each sublayer is still large enough to do its task at the same level as the full layer. This has precedent in the cognitive psychology literature, where, for example, subjects being tested on recall of a list over time or in different contexts may be allowed as many trials as they need to memorize the list in the first place, so that all participants start out with the same low training error rate. This assumes that humans have enough neurons available to memorize the training list to whatever degree of accuracy is required, given enough time. In addition, it is known that in rats, during the course of a particular spatial task, only a small fraction of the hippocampal CA1 neurons fire during the entire duration of the task. This suggests that the hippocampus has many more neurons than necessary for any given task.

Of course, neurons cannot be added to actual test subjects, but in our test networks this provides an effective way to accomplish the same goal of reducing the error on the training set, so that all networks start with the same baseline error rate. From the biological standpoint, this way of comparing networks essentially says that, in the actual brain, the memorization ability of the hippocampus for any particular task is not limited by the number of neurons, but rather the way in which they are connected. From this point of view, the basic AllSplit and SplitDG networks should be ignored, and the results of Baseline should only be compared against AllSplit+ and SplitDG+, since all three of these networks have the same error rate on the initial training set.

The second way to interpret the results is to take the neuron-limited view, where a fair comparison would be between networks which have the same number of neurons, regardless of how well they are able to store the initial training set. In this view, splitting a layer into two separate sublayers incurs the penalty of each sublayer now being half the size. Biologically, this means that neurons are costly in terms of energy required to build and maintain, and that the brain has as few neurons as possible while still being able to perform its required tasks. From this point of view, Baseline should be compared with AllSplit and SplitDG since they have the same number of neurons, and AllSplit+ and SplitDG+ should be ignored.

**Figure 14:**
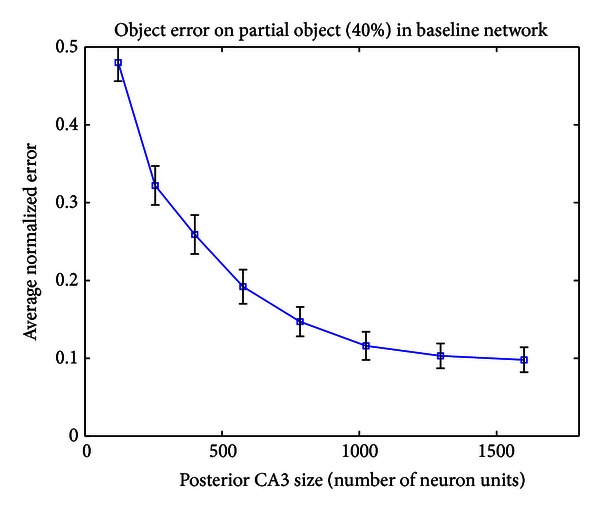
Error on object output layer in a 40% partial object task as a function of the size of posterior CA3 in the Baseline network.

In the biological hippocampus, the answer probably lies somewhere between the two extremes.  Figure 14 shows that increasing the size of CA3 in the Baseline network results in lower error rates, but that eventually the error stops decreasing with layer size. If the hippocampus is in the rightmost region of the graph, then it has enough neurons and there is little cost to splitting a layer, so it is best approximated by the “+” models. On the other hand, if it is near the leftmost region of the graph, it is severely neuron constrained, and splitting a layer results in a dramatic decrease in performance on each of the streams. In this case it would be better approximated by the normal (non-+) models.

Overall, the test results show that the AllSplit network is best for noisy or partial object situations and worst when given noisy or partial context. AllSplit+ has uniformly better performance as expected but follows the same general pattern as AllSplit. On the other hand, the Baseline network is relatively better at noisy or partial context situations than with noisy or partial object. Rarely is it the best network at any particular task, however, with the exception of partial context. It is most similar to the SplitDG network, which is what we expected based on its architecture. The SplitDG network has good all-around performance. Compared to Baseline, it does consistently better in noisy or partial object tests, about the same in noisy context, but noticeably worse when presented with partial context. SplitDG+ is generally about the same as SplitDG on noisy or partial object tests, but its larger DG seems to aid in the incorporation of context information when it is noisy or partial. This allows it to do significantly better than SplitDG in such tasks and puts it on par or better than Baseline. Our results thus suggest that differentiation within DG provides uniformly better performance over a nondifferentiated DG if it is large enough (SplitDG+), and generally better performance with the exception of partial context tasks if DG is size constrained (SplitDG). Additional differentiation within CA3 (AllSplit and AllSplit+) may work to further increase noisy and partial object task performance, but at the cost of the corresponding degraded context task performance.

### 4.3. Object Noise versus Context Noise

These results raise the question of whether it is better for the object stream to be able to deal with noisy objects (AllSplit) or noisy contexts (Baseline), where we will use the term “noise” to refer to partial cues as well. We argue that there is inherently less noise in contexts than in objects; hence dealing with object noise is more important. To make things concrete, consider the case of an animal in search of food. It has to find edible plants and insects and has to memorize a large amount of object-related information. Depending on the time of year and the time of day, the types of plants or insects it can eat and their appearance change (noise). On the other hand, the season and spatial environment are contextual cues that change slowly, and there are only a relatively small number of different contexts it must identify: its dwelling, its scavenging grounds, what season it is, and so forth. In general, the much larger number of objects in existence makes it likely that interference and noise are much more likely to occur between objects than between object and contexts, which are few in number and change only slowly over time. 

The second argument is that, given some recurrent support structures, noise in context is easier for the hippocampus to deal with than noise in object. The context stream deals with context noise relatively well since the contexts are few and well memorized. Thus getting a clean context to the object stream requires only taking the context stream output (C) and feeding it back into the object stream. If the context is very noisy or absent (to the point that the context stream output is no longer useful), the output of the OBCG layer can be used instead. Thus there are two independent ways for the object stream to not have to deal with context noise, each involving only a recurrent loop.

With object noise, the situation is different. The object stream is itself responsible for determining the object; thus the only place it can turn to for additional object information is the CBOG output, which uses context to make object guesses. However, since the CBOG stream uses mainly context information, the best it can do is to give a list of possible objects that are associated with that context. Choosing one object out of this list would then require a separate calculation where the input object is compared with the CBOG output list and the best match selected. This would not be an easy task when the input object is noisy, although it would be significantly easier than the object stream's original task, which is to compare the input object to a list of 120 possible objects and choose the closest match. Thus the object noise problem can certainly be overcome with the help of additional structures, but it may be more judicious to simply use context information in the object stream from the beginning, which is exactly the solution that the AllSplit and SplitDG networks use. They then trade the object noise problem for a context noise problem, but this seems to be a much easier issue to deal with.

### 4.4. Mismatches

Mismatches, consisting of an object appearing in a different context from that it was learned into, are by definition rare events. If they happened frequently, the object would simply be associated with the new context and it would no longer be considered a mismatch. On the posterior side, a mismatch means that the incoming context information does not match the primary object input from LEC, thus putting it in a situation similar to having a very noisy context but noiseless object. On the anterior side, where MEC context information is primary, the incoming object input introduces uncertainty, and the situation is similar to a very noisy object but noiseless context. Due to the smaller number of inputs it needs to store and the fact that LEC input is relatively weak, mismatches have little effect on the anterior stream—if we see someone from the office at the mall, we do not have any trouble recognizing our context as the mall. On the other hand, the large amount of MEC input into the posterior stream means that a mismatched context can significantly affect object recognition—it may take us several seconds to recognize a colleague if we unexpectedly encounter them at the mall, whereas the recognition is nearly instantaneous when we see them at the office. 

Any encoding and retrieval scheme which uses contextual information to recognize objects, as we believe the hippocampus does, will naturally have problems in mismatch situations. However, this is only the case if we believe that a familiar object in a different context from usual ought to still be recognized as the same familiar object. In many situations it may make sense to consider object A in context A as effectively different from object A in context B [42]. The large amount of error that a mismatch produces may be beneficial for signaling that something is wrong or unexpected and deserves our attention.

### 4.5. Relation to Rat Hippocampus

Our model is not explicitly a place field model, and in the way we have conceptualized it and in its current form our model better reflects the primate hippocampus. However, with some minor modifications the model would be consistent with the observation of higher-resolution place fields in dorsal compared to ventral hippocampus. We will switch to using the appropriate terminology for the rat anatomy in this discussion, so that anterior and posterior in our model are now ventral and dorsal, and the caudolateral and rostromedial bands of MEC and LEC are now dorsolateral and ventromedial, respectively.

In our model, for simplicity's sake, we make no distinction between the dorsolateral and ventromedial bands of the MEC, modeling both as carrying the same context information, albeit to different parts of hippocampus (dorsal versus ventral, resp.). However, it is known that neurons in the dorsolateral band of MEC are more spatially tuned than those in the ventromedial band [43], and thus we would expect that the dorsal hippocampus, receiving higher-resolution spatial information from the dorsolateral band, would have the tighter place fields that are seen experimentally. If we wanted to extend our model to cover this additional aspect of the anatomy, we could do this by having two different types of contextual inputs, a “local” context and a less precise “global” context which might represent the context at a larger spatial scale or contain some other nonspatial information, with the local context being carried by the dorsolateral MEC and the global context being carried by the ventromedial MEC. 

Note that both the dorsal and ventral subdivisions of the hippocampus receive the nonspatial LEC inputs to some extent. However, we refer to the dorsal hippocampus as the more object-oriented layer in our model compatible with human fMRI studies and our set of sample tests (shown in Figures 6 and 7) which led us to set the relative weighting of the LEC input larger than that of the MEC input for optimal performance (and the reverse is true on the ventral hippocampus for context information). Of course, the set of “tests” that the rat hippocampus has evolved to do could be different from the basic tests that we proposed. For example, the performance on the mismatch test (where the presented object and context were not associated) was a significant factor in determining how strong the MEC to dorsal hippocampus connections should be. A strong MEC to dorsal connection results in a large amount of error on the OBCG output, and as a result those connections were kept very weak. In the rat hippocampus, however, it could be the case that it simply just does badly on mismatches because they are so rare that they do not need to be protected against with weak MEC to dorsal weighting, or it could be that in the case of mismatches, additional cortical processing is involved. In either case, the MEC to dorsal signal could well be just as strong or stronger than the LEC to dorsal signal. 

In conjunction with the dorsolateral versus ventromedial band differences mentioned above, the dorsal and ventral streams of our rat-modified model would not contradict the general conception of the dorsal stream as being context oriented and more finely spatially tuned than the ventral side. In summary, the degree to which the MEC's spatial contextual information is relevant in the dorsal side of the rat hippocampus is probably much higher than that indicated in our model, where we look at objects, rather than context, as the primary information the hippocampus is storing and view context as information that can contribute to object recognition.

## 5. Conclusion

We constructed hippocampus models that include anatomical and functional details such as the distinction between the posterior and anterior subdivisions of the hippocampus, connections from the medial and lateral entorhinal cortex to both the posterior and anterior regions, differences between the superior and inferior blades of the dentate gyrus, and connectivity differences between distal and proximal (relative to DG) portions of CA3 and CA1. We hypothesized distinct roles for each of the CA1 areas on the proximal and distal sides and attempted to show how these anatomical details work together to increase performance on certain tasks. In particular, we showed that object and context require different treatment in terms of how much one is used to help recognize the other. This is simply due to the greater number of objects compared to the number of contexts rather than intrinsic differences in representation. In addition, we showed how the hippocampal anatomy supports the use of contextual information to help object recognition and proposed ways in which the tradeoffs inherent to this could possibly be mitigated. 

Our models make several predictions that may be experimentally tested. We predict that the inferior blade of DG and proximal CA3 in the posterior region of hippocampus receives more MEC innervation, or that these neurons are more sensitive to MEC inputs, than is the case with LEC inputs into the anterior side of hippocampus. Blocking MEC input into posterior hippocampus should have a significant negative effect on object recognition when the object is noisy or only partially shown, assuming that the object was associated with a specific context, but should have only a mildly negative or even a positive effect if the context is noisy or obscured. Blocking LEC input into anterior hippocampus should have much less of an effect on context recognition in either case, assuming that there are many more objects than contexts. If the number of contexts and the number of objects are roughly equal, then we should see effects similar to those seen on the posterior stream with MEC input. Our assumptions about the two different types of information being carried along the output pathways can also be experimentally tested by comparing the information content of proximal CA1 and distal CA1 neurons. We predict that distal CA1 neurons on both the posterior and anterior sides will be more likely to carry object-type information, while proximal CA1 neurons will tend to carry primarily context-type information.

We found that the models that have only DG split (SplitDG and SplitDG+) did the best overall on our test sets, generally doing about the same as the Baseline model when the context input was degraded, and significantly better when the object input was degraded. The models with both DG and CA3 split (AllSplit and AllSplit+) did even better in noisy or incomplete object situations, but at a cost in performance on the corresponding degraded context tasks. As we mentioned in the discussion, it may be the case that degraded context situations are relatively rare compared to degraded object situations, and thus the performance tradeoff of the AllSplit networks may in fact be optimal. However, it is probably also the case that the hippocampus does not make as severe a tradeoff as we have in our models, where CA3 is either completely unified or completely split. For instance, both regions of CA3 in the actual hippocampus receive superior blade input from DG, rather than just the distal region. In our model, the superior blade on the posterior side of hippocampus carries mainly LEC object information, so including this feature may change the ratio of object to context information within proximal CA3 in favor of object information and thereby reduce some of the deleterious effects of noisy context that we observed in the AllSplit network. The two regions of CA3 also communicate to an extent, although they have different connectivity patterns in terms of the proportion of projections they send within CA3 and onward to CA1. Exactly how these differences affect hippocampal function remains a topic for future research.

To date, much of the computational literature on the hippocampus has either focused on only object memorization or only spatial context memorization and has not attempted to identify how these different types of information may mutually support each other within the hippocampus or elucidate specific anatomical details within the hippocampus that may allow this to occur. On the other hand, experimental literature that addresses details such as the LEC and MEC crossconnections has often assigned them only the vague role of allowing a mixing or integration of object and context information. We have hypothesized specific ways that object and context information may be used in the posterior and anterior regions of the hippocampus, shown that the connectivity of hippocampus supports and enables these uses, and identified specific situations in which these object-context interactions have a beneficial or deleterious effect. Our results thus suggest new ways of thinking about the sort of computations that the hippocampus may do, and how it uses both object and context to perform them.
